# Commentary: Gut Microbiota-Related Evidence Provides New Insights Into the Association Between Activating Transcription Factor 4 and Development of Salt-Induced Hypertension in Mice

**DOI:** 10.3389/fcell.2021.654447

**Published:** 2021-04-07

**Authors:** Kun Zhao, Yukang Mao, Wei Sun

**Affiliations:** Department of Cardiology, The First Affiliated Hospital of Nanjing Medical University, Nanjing, China

**Keywords:** salt-sensitive, salt-resistance, hypertension, gut microbiota, salt diets

## Introduction

We recently read with interest the recent article by Liu et al. (2020) regarding the pivotal regulation of activating transcription factor 4 (ATF4) in gut microbiota that is highly associated with the development of high-salt diet-induced hypertension in mice. However, this article lacks the comparisons of ATF4 and gut microbiota among salt-sensitive, salt-resistant, and control mice. In this commentary, we will emphasize the importance of incorporating salt-resistant mice into the study.

## Subsections Relevant for the Subject

After transcriptomic and metabolomic analysis, ATF4 was reported to mediate cellular metabolism reprogramming by inducing cellular inflammation, apoptosis, and mitochondrial dysfunction which may trigger the integrated stress response via activating the genes involved in the mitochondrial stress, and the gut microbiome, suggesting the vital role of ATF4 in metabolism-related diseases (Quirós and Prado, 2017; Wu et al., 2020; Xu et al., 2020). Besides, ATF4 was found to enhance endothelial inflammatory response by targeting hypertension-related miR-1283 (He et al., 2016). It is also noteworthy that ATF4 may affect the intestinal microenvironment closely related to hypertension through directly regulating the production of gut microbial metabolites (Hu et al., 2019; Touyz and Camargo, 2019), thereby providing the theoretical basis for its involvement in hypertension. Thus, Liu et al. performed fecal microbiota transplantation (FMT) in the *Atf4* knockout (KO) or overexpression mice to explore whether ATF4 could play a critical role in high-salt diet-induced hypertension and vascular endothelial dysfunction. The article demonstrates that activated ATF4 participated in the high-salt diet-induced elevated blood pressure (BP) by regulating gut microbiota composition and vitamin K2 (VK2) synthesis, shedding light on the novel mechanism underlying the effects of ATF4 on high-salt diet-induced hypertension from the perspective of intestinal metabolomics. To be noted, another two independent studies by Bier et al. (2018) and Yan et al. (2020) also identified that the high-salt diet, which interfered with the normal gut's microbial composition, could exert an adverse regulatory effect on normal BP by regulating metabolites. Collectively, these findings provide robust evidence that the reshaped intestinal bacteria profile under the context of the high-salt diet gives rise to hypertension.

As an immense global concern that increases the risk of mortality, hypertension is a pathological status characterized by persistent high BP of which the underlying mechanism remains obscure. The robust detailed analysis of a large multi-population-based study first indicated that salt intake is the most important factor in determining the BP level (He et al., 2013). Moreover, present data has confirmed the positive correlation between high-salt intake and increased BP (Ritz, 2010).

Research in recent years found that the incidence of salt-sensitive or salt-resistant rat or mice models are different when the high-salt diet begins at different body weight or age. In addition, the prevalence of salt-sensitivity or salt-resistance in rodent models is closely associated with the treating duration of the high-salt diet (Balafa and Kalaitzidis, 2021). Due to the existence of salt sensitivity and resistance, albeit different individuals may show different BP responses to dietary salt intake, the results of numbers of prospective cohort studies and retrospective studies showed that excessive salt intake (>4.6 g/day) can increase the incidence of adverse cardiovascular events, including cardiac interstitial and perivascular fibrosis, myocardial hypertrophy, and even heart failure, in both hypertensive and normotensive populations regardless of age and race (Strazzullo et al., 2009), indicating that high-salt diet independent of BP is an independent risk factor for cardiovascular disease.

## Discussion

However, Liu et al. did not mention the presence of normotensive mice after a high-salt diet feeding. More importantly, their study lacked a specific indication of the proportion of salt-resistant and salt-sensitive mice. From all the above, it may further strengthen the rationality of the conclusion that the impact of high-salt intake alone on intestinal flora contributes to the elevated BP if Yan et al. could also compare the differences of ATF4 expression, and then perform 16S rRNA gene sequencing of intestinal flora among salt-sensitive, salt-resistant, and control mice, respectively.

Moreover, concerning the fact that moderate control and reduction of salt intake can ameliorate the high-salt diet-induced risk of cardiovascular disease (Wilson et al., 2019), we also suggested Yan et al. to consider switching the mice to a normal diet after a 4-week high-salt diet, and then observing whether the altered ATF4 expression and intestinal flora could return to normal status, so as to determine the changes of ATF4 and intestinal flora caused by high salt diet are either reversible or irreversible.

## Author Contributions

All authors listed have made a substantial, direct and intellectual contribution to the work, and approved it for publication.

## Conflict of Interest

The authors declare that the research was conducted in the absence of any commercial or financial relationships that could be construed as a potential conflict of interest.
